# RENAL INJURY AND METABOLIC DYSFUNCTION-ASSOCIATED STEATOTIC LIVER DISEASE IN PATIENTS WITH OBESITY

**DOI:** 10.1590/S0004-2803.24612025-008

**Published:** 2025-05-02

**Authors:** Kellyane Dias CARVALHO, Cláudia DALTRO, Carla DALTRO, Helma Pinchemel COTRIM

**Affiliations:** 1 Universidade Federal da Bahia, Faculdade de Medicina da Bahia, Programa de Pós-Graduação em Medicina e Saúde, Salvador, BA, Brasil.

**Keywords:** Metabolic dysfunction-associated steatotic liver disease, steatohepatitis, renal injury, metabolic surgery, Doença hepática gordurosa associada à disfunção metabólica, esteato-hepatite, lesão renal, cirurgia metabólica

## Abstract

**Background:**

Metabolic dysfunction-associated steatotic liver disease (MASLD) is currently the most prevalent cause of chronic hepatic disease worldwide. Recently, the association between MASLD and renal injury has emerged as an additional factor impacting the clinical course of MASLD.

**Objective:**

The present study evaluated the clinical association in patients with obesity.

**Methods:**

This study enrolled patients classified as having obesity class II and III (BMI >35 kg/m^2^) and MASLD from an obesity surgical treatment center. The diagnosis criteria for MASLD included the presence of hepatic steatosis as indicated by histology or imaging assessments. We use Fibrosis-4 (FIB-4) and NAFLD fibrosis score (NSF) to assess and determine the presence of liver fibrosis. The glomerular filtration rate (GRF) was determined using CKD-EPI (chronic kidney disease epidemiology collaboration) equation, with GFR levels ≥90 and <120 mL/min/1,73 m^2^ considered within the normal range.

**Results:**

The study comprised a total of 560 individuals with obesity grade II and III, 325 individuals with MASLD. Among these, 422 (75.4%) patients were female, and the mean age was 36±10 years. Systemic arterial hypertension (SAH) was present in 162 (41.1%) patients, and 218 (42.8 %) were diagnosed with type 2 Diabetes Mellitus (T2DM). A total of 286 individuals (51.1%) had a GFR below 114 mL/min, with 183 (64%) of them exhibiting a higher degree of liver fibrosis, as indicated by FIB-4 >0.54.

**Conclusion:**

In patients with obesity classified as grades II and III, age emerged as the primary determinant leading to decline in GFR. Furthermore, glomerular hyperfiltration could be an early sign of progression to chronic kidney disease. Nonetheless, the progression of hepatic fibrosis could also be a significant factor contributing to impaired renal function.

## HIGHLIGHTS

• The association between MASLD and renal injury has emerged as an additional factor impacting the clinical course of MASLD.

• Glomerular hyperfiltration may be an early indicator of chronic kidney disease progression.

• Progression of hepatic fibrosis may be a significant factor contributing to impaired renal function.

## RESUMO

**Contexto:**

A Doença hepática gordurosa associada à disfunção metabólica (DHGADM) é atualmente a causa mais prevalente de doença hepática crônica no mundo. Recentemente, a associação entre DHGADM e injúria renal surgiu como um fator adicional que impacta o curso clínico da DHGADM.

**Objetivo:**

O presente estudo avaliou a associação clínica em pacientes com obesidade.

**Métodos:**

Este estudo incluiu pacientes classificados com obesidade de classe II e III (IMC >35 kg/m²) e DHGADM, de um centro de tratamento cirúrgico para obesidade. Os critérios diagnósticos para DHGADM incluíram a presença de esteatose hepática indicada por histologia ou exames de imagem. Utilizamos o Fibrosis-4 (FIB-4) e o escore de fibrose NAFLD (NSF) para avaliar e determinar a presença de fibrose hepática. A taxa de filtração glomerular (TFG) foi determinada usando a equação CKD-EPI (*chronic kidney disease epidemiology collaboration*), com níveis de TFG ≥90 e <120 mL/min/1,73 m² considerados dentro do intervalo normal.

**Resultados:**

O estudo incluiu um total de 560 indivíduos com obesidade de grau II e III, sendo 325 com DHGADM. Entre estes, 422 (75,4%) eram mulheres, e a idade média foi de 36±10 anos. Hipertensão arterial sistêmica (HAS) estava presente em 162 (41,1%) pacientes, e 218 (42,8%) foram diagnosticados com Diabetes Mellitus tipo 2 (DM2). Um total de 286 indivíduos (51,1%) apresentou TFG abaixo de 114 mL/min, com 183 (64%) deles exibindo um grau mais elevado de fibrose hepática, conforme indicado por FIB-4 >0,54.

**Conclusão:**

Em pacientes com obesidade classificado como graus II e III, a idade surgiu como o principal determinante para o declínio na TFG. Além disso, a hiperfiltração glomerular pode ser um sinal precoce de progressão para doença renal crônica. No entanto, a progressão da fibrose hepática também pode ser um fator significativo que contribui para a função renal comprometida.

## INTRODUCTION

Metabolic dysfunction-associated steatotic liver disease (MASLD) is estimated to affect up to 30% of the adult population around the world^1^. MASLD encompasses a wide spectrum of disorders, ranging from simple steatosis to steatohepatitis, which can progress to hepatocellular carcinoma and liver cirrhosis^2^. MASLD also is a multisystem condition with extra-hepatic involvement that affects not only the liver but also the cardiovascular system and kidneys^1^, making it a leading cause of liver transplantations.

Emerging clinical research suggest a profound link between hepatic steatosis and renal dysfunction. These studies elucidate that the severity of liver disease correlates with the risk of developing kidney complications, positioning MASLD/MASH within that adversely affect renal health^.^ Renal complications are integral to the systemic impact of MASLD/MASH, underscoring the complex interplay of metabolic disorders^3^. 

Recent studies suggest that factors such as metabolic syndrome, dysbiosis, platelet activation, and aging may play a role in the connection between kidney damage and MASLD^3^. Furthermore, evidence suggests the presence of patatin-like phospholipase domain-containing protein 3 (PNPLA3), which is associated with an increased susceptibility to MASLD. Additionally, alcohol consumption may contribute to the decline in GFR in these patients^4^.

Previous studies have reported CKD in 20-25% of individuals with MASLD^5^. Considered the substantial prevalence of MASLD, further insights into the subject are highest clinical relevance for establishing primary prevention strategies for CKD. 

This ongoing research is a part of a larger investigation conducted in Brazil, focusing on patients with obesity and MASLD^6^ ^-^ ^8^ ^.^Its aim is to assess the clinical relevance of renal dysfunction in these patients. 

## METHODS

### Study design and population

This descriptive study used secondary data derived from a previous project that evaluated MASLD in individuals with obesity undergoing treatment at a specialized obesity treatment center. They had any recommendations regarding lifestyle changes accompanied by the nutritionist.

All study participants with obesity grade 2 or higher were included in our database the day before their bariatric surgery. 

### Inclusion criteria

Individuals with a BMI ≥35 kg/m^2^, who had undergone bariatric surgery in accordance with CDC criteria^9^. This investigation was part of a project entitled “hepatic disease in the severely obese”, which had previously obtained approval from our institution’s Ethics and Research Committee. All included patients provided written informed consent, and the present research protocol was approved by the Institutional Review Board of our institution.

### Exclusion criteria

Individuals undergoing kidney replacement therapy, had symptomatic cardiovascular disease, taking medications known to reduce kidney function within the last three months (glucocorticoids, diuretics, and nonsteroidal anti-inflammatory drugs), diagnosed with chronic viral hepatitis B (HBsAg negative) or C anti HCV negative), exceeding international alcohol consumption guidelines (21 g/day for men and 14 g/day for women), or having other chronic liver diseases were excluded from the study. All patients were HIV negative.

**Clinical evaluation:** the data were extracted from patient medical records and included information on age, gender, medical history, alcohol consumption, medication usage, BMI and exposure to environmental toxins. Biochemical assessments encompassed a liver panel, featuring serum levels of AST (aspartate aminotransferase), ALT (alanine aminotransferase), GGT (gamma-glutamyl transferase), alkaline phosphatase, bilirubin, prothrombin time, total cholesterol and its fractions, total proteins and their fractions, fasting blood glucose, triglycerides, viral markers for hepatitis B and C viruses, serum insulin, transferrin saturation, and ferritin).

The metabolic syndrome definition followed the guidelines of the International Diabetes Federation^10^. Dyslipidemia criteria: triglycerides ≥150 mg/dL, low HDL cholesterol (<50 mg/dL for women and <40 mg/dL for men); or statin or fibrate use, irrespective of the level of serum lipids. The Insulin Resistance Assessment Model (HOMA-IR) was used to assess insulin resistance. The definition of insulin resistance^11^ was an index value ≥3. Systemic arterial hypertension was defined as values ≥130x85mmHg or treatment for hypertension^1^. All participants underwent abdominal ultrasonography. 

### Definition of MASLD

Metabolic dysfunction-associated steatotic liver disease (MASLD), previously termed non-alcoholic fatty liver disease (NAFLD), is defined as steatotic liver disease (SLD) in the presence of one or more cardiometabolic risk factor(s) and the absence of harmful alcohol intake^1^.

### Definition of obesity

These evaluations were performed according to the Centers for Disease Control and Prevention (CDC) guidelines. Obesity Class I obesity: BMI 30-35 kg/m²; Class II: BMI: 35-40 kg/m² and Class III: BMI>40 kg/m². In specific situations, it could be categorized as “severe obesity”^9^. 

Type 2 diabetes mellitus (T2DM) was defined in accordance with the American Diabetes Association (ADA)^12^: glycated hemoglobin (A1C ≥6.5%) or fasting plasma glucose (FPG ≥126 mg/dL) or 2-h plasma glucose (PG ≥200 mg/dL) during oral glucose tolerance test (OGTT) or in an individual with classic symptoms of hyperglycemia or hyperglycemiccrisis, a random plasma glucose ≥200 mg/dL. In the present study, dysglycemia was defined as glucose alterations > 100 mg/dl in patients who had not undergone an evaluation for T2DM.

### Assessment of hepatic fibrosis

The non-invasive markers of liver fibrosis,FIB-4^13^ ^-^ ^15^ and NSF score^16^ ^,^ ^17^, were used to define the presence or absence of liver fibrosis.

### Assessment of GFR

Estimated GFR (eGFR) was assessed according to the chronic kidney disease epidemiology collaboration (CKD-EPI)^18^ ^,^ ^19^. 

Systemic arterial hypertension (SAH) was defined in accordance with the Brazilian Cardiology Society^20^: PA ≥140/80 mmHg or on therapy for hypertension.

### Statistical analysis

The analyzes and data tabulation were carried out using SPSS (Statistical Package for Social Science). Categorical data were presented in terms of simple, relative, and absolute frequencies, while quantitative data were reported as mean and standard deviation. For comparison purposes, the subjects were split into two groups based on median glomerular filtration rate (GFR, ml/min). Pearson’s Chi-square test was utilized to compare groups, and independent samples were compared using Student’s *t*-test. The influence of the variables studied on the GFR was analyzed by multivariate regression. The model incorporated the following variables: age, gender, arterial hypertension, diabetes mellitus, dyslipidemia, and FIB-4. The latter was categorized into two groups according to the median (0.54). *P* values <0.05 were deemed statistically significant.

## RESULTS

This study included 560 individuals with grade II and III obesity. 

Table 1 summarizes the clinical characteristics and their respective GFR in patients with obesity and without MASLD.

**TABLE 1:** Clinical characteristics of 560 individuals with obesity class II and III, total and according to glomerular filtration rate. Salvador-Bahia-Brazil, 2015-2018.

Characteristics	Total 560 (100%)	Glomerular filtration rate		*P*	*P* adjusted*
		≤114	>114		
		286 (51.1%)	274 (48.9%)		
Age (mean and standard deviation)	36 (10)	41 (10)	31 (7)	**<0.001**	<0.001
Sex				**0.024**	0.100
Male	138 (24.6%)	59 (20.6%)	79 (28.8%)		
Female	422 (75.4%)	227 (79.4%)	195 (71.2%)		
Body mass index	41.0 (5.0)	40.9 (5.0)	40.9 (5.0)	0.892	-
Arterial Hypertension	218 (42.8%)	130 (48.7%)	88 (36.4%)	**0.005**	0.948
Diabetes Mellitus	88 (16.0%)	57 (20.4%)	31 (11.5%)	**0.005**	0.893
Dyslipidemia	264 (47.3%)	143 (50.4%)	121 (44.2%)	0.143	0.819
Steatosis	325 (58.1%)	170 (59.4%)	155 (56.8%)		
Weight loss treatment	67 (12.0%)	33 (17.5%)	34 (18.3%)	0.836	-
FIB-4 score				**<0.001**	0.645
> 0.54	278 (49.8%)	183 (64.0%)	94 (34.3%)		
≤0.54	280 (50.2%)	103 (36.0%)	180 (65.7%)		

*Variables included in the multivariate regression model: age, sex, arterial hypertension, diabetes mellitus, dyslipidemia and FIB-4 score.

Out of the total participants, 422 (75.4%) were female. The mean age of the patients was 36±10 years, and it was higher in the group with the lowest GFR. The average BMI was 41±5 kg/m². DM2 was diagnosed in 218 (42.8%) individuals. SAH) and dyslipidemia were observed in 162 (41.1%) and 264 (47.3%) individuals, respectively.

Based on the categorization of the mean GFR, 286 individuals (51.1%) had a GFR lower than 114 mL/min. Of these, 183 (64%) had a higher degree of liver fibrosis with FIB-4>0.54.

## DISCUSSION

This study demonstrated that, in patients with grade II and III obesity, age was associated with a more pronounced decrease in glomerular filtration rate (GFR), and MASLD was not responsible for the decline in renal function. The patients exhibited a high glomerular filtration rate. These patients had a relatively young average age, and most of them did not have metabolic syndrome or hypertension. Nevertheless, they did not show a higher level of liver fibrosis.

Recent clinical studies have identified MASLD/MASH as independent predictors for both the onset and progression of kidney disease, suggesting a relationship between hepatic steatosis and renal dysfunction. In this context, the severity of liver disease correlates with the risk of developing renal complications^21^. 

High glomerular filtration rate could be justified because these patients had obesity. Abbate et al. identified that patients with MASLD presented higher levels of eGFR as well as a significantly increased prevalence of hyperfiltration (73.2%). MASLD and increased weight were associated with an increased probability of presenting hyperfiltration^22^.

Establishing an association between MASLD and glomerular hyperfiltration could aid in the early identification of patients at increased risk of chronic kidney disease (CKD). It is suggested that individuals with MASLD be screened for GFR or urinary albumin excretion. However, since hyperfiltration often precedes CKD, screening for abnormally high GFR could facilitate even earlier recognition of potential risk^23^.

Sirota and colleagues carried out an extensive cross-sectional investigation involving 11,469 adults who engaged in the National Health and Nutrition Examination Survey from 1988 to 1994, known as NHANES III^24^. Their hypothesis revolved around the potential association between MASLD and CKD, with a particular focus on whether the severity of liver disease raised the CKD risk. Their findings suggested a correlation between the presence and severity of MASLD and CKD. However, this association weakened after adjusting for confounding factors^24^.

Nevertheless, the ongoing discussion highlights that the progression of liver fibrosis plays a pivotal role in elevating the risk of CKD. Zuo and colleagues revealed that, among individuals with MASLD, the progression of liver fibrosis stands out as the most significant predictor for the development of renal disease, compared to baseline levels of metabolic disease. Notably, markers of fibrosis offer valuable risk stratification, aiding in the identification of MASLD patients at a bigger risk of kidney-related issues^25^. 

In the context of liver steatosis, the European Association for the Study of the Liver recommends the assessment of serum marker for fibrosis^26^. The NFS (NAFLD Fibrosis Score) is a useful in identifying individuals with a lower risk of progressing to advanced fibrosis and has undergone external validation in ethnically diverse MASLD populations^15^ ^,^ ^27^.

This study has limitations. Firstly, it relied just on the estimation of GFR to assess renal function, without considering other markers such as albuminuria. However, it is important to note that GFR is the most used variable in epidemiological studies for assessing CKD and it is used to classifying its stages, making the findings of this study still relevant and valuable. 

In conclusion, among patients with obesity grades II and III, age emerged as the primary factor associated with the decline in GFR. However, the progression of hepatic fibrosis could also be a significant role in impaired renal function. Furthermore, glomerular hyperfiltration could be an early sign of progression to chronic kidney disease. These results underscore the importance of monitoring MASLD though ultrasound and longitudinal non-invasive fibrosis markers to identify individuals at risk of early progression to chronic kidney disease. 
